# Artificial Intelligence Applied to *in vitro* Gene Expression Testing (IVIGET) to Predict Trivalent Inactivated Influenza Vaccine Immunogenicity in HIV Infected Children

**DOI:** 10.3389/fimmu.2020.559590

**Published:** 2020-10-05

**Authors:** Nicola Cotugno, Veronica Santilli, Giuseppe Rubens Pascucci, Emma Concetta Manno, Lesley De Armas, Suresh Pallikkuth, Annalisa Deodati, Donato Amodio, Paola Zangari, Sonia Zicari, Alessandra Ruggiero, Martina Fortin, Christina Bromley, Rajendra Pahwa, Paolo Rossi, Savita Pahwa, Paolo Palma

**Affiliations:** ^1^Academic Department of Pediatrics (DPUO), Research Unit of Congenital and Perinatal Infections, Bambino Gesù Children's Hospital, Rome, Italy; ^2^Chair of Pediatrics, Department of Systems Medicine, University of Rome “Tor Vergata”, Rome, Italy; ^3^Miami Center for AIDS Research, Department of Microbiology and Immunology, Miller School of Medicine, University of Miami, Miami, FL, United States; ^4^Academic Department of Pediatrics (DPUO), Research Unit of Growth Disorders, Bambino Gesù Children's Hospital, Rome, Italy; ^5^BioStat Solutions, Inc., Frederick, MD, United States

**Keywords:** gene expression, predictive biomarkers, artificial intelligence, deep learning, influenza vaccine, HIV, vaccinomics

## Abstract

The number of patients affected by chronic diseases with special vaccination needs is burgeoning. In this scenario, predictive markers of immunogenicity, as well as signatures of immune responses are typically missing even though it would especially improve the identification of personalized immunization practices in these populations. We aimed to develop a predictive score of immunogenicity to Influenza Trivalent Inactivated Vaccination (TIV) by applying deep machine learning algorithms using transcriptional data from sort-purified lymphocyte subsets after *in vitro* stimulation. Peripheral blood mononuclear cells (PBMCs) collected before TIV from 23 vertically HIV infected children under ART and virally controlled were stimulated *in vitro* with p09/H1N1 peptides (stim) or left unstimulated (med). A multiplexed-qPCR for 96 genes was made on fixed numbers of 3 B cell subsets, 3 T cell subsets and total PBMCs. The ability to respond to TIV was assessed through hemagglutination Inhibition Assay (HIV) and ELIspot and patients were classified as Responders (R) and Non Responders (NR). A predictive modeling framework was applied to the data set in order to define genes and conditions with the higher predicted probability able to inform the final score. Twelve NR and 11 R were analyzed for gene expression differences in all subsets and 3 conditions [med, stim or Δ (stim-med)]. Differentially expressed genes between R and NR were selected and tested with the Adaptive Boosting Model to build a prediction score. The score obtained from subsets revealed the best prediction score from 46 genes from 5 different subsets and conditions. Calculating a combined score based on these 5 categories, we achieved a model accuracy of 95.6% and only one misclassified patient. These data show how a predictive bioinformatic model applied to transcriptional analysis deriving from *in-vitro* stimulated lymphocytes subsets may predict poor or protective vaccination immune response in vulnerable populations, such as HIV-infected individuals. Future studies on larger cohorts are needed to validate such strategy in the context of vaccination trials.

## Introduction

The advent of vaccinations has reshaped the history of medicine and across the twenty-first century has led to a decrease in morbidity of previously fatal diseases (1, 2). However, with steadily improving survival rates due to the availability of novel therapeutic tools, the vulnerable populations with special vaccination needs is burgeoning (3–5). Nowadays vaccine development programs mainly focus on otherwise healthy populations; as such, vaccine indications are based on data arising from healthy study participants. Accordingly, most vaccine indications in vulnerable groups (VPs), elderly, pregnant women and patients affected by chronic conditions (i.e., HIV infected patients), are derived from extrapolations, assumptions, or post-licensure studies (5). Thus, limited data are currently available to tailor vaccine interventions in these populations. Since the seasonal flu vaccine is well-established in routine use in HIV, it may represent the paradigm vaccine to illustrate many of the issues that affect most or all vaccines in VPs. Despite recommendations on seasonal influenza vaccination for targeted or at-risk groups (i.e., HIV, elderly, comorbidities etc.), such populations are at increased risk of acquiring vaccine-preventable infections and suffer higher infectious morbidity and mortality than healthy individuals (6, 7). This represents a major health and economic burden to society, which will become increasingly difficult to manage given limited public resources (8). In parallel, many uncertainties remain about the optimal strategies for identifying susceptible individuals, and for offering them sustained protection through a personalized immunization schedule. Novel biomarkers of protective immune responses to vaccines are needed. Vaccinology, based on the immune response network theory (9), which utilizes immunogenetics, immunogenomics and systems biology approaches to understand the basis for inter-individual variations in vaccine induced immune responses can provide such biomarkers (10, 11). In particular, vaccinomics utilize high-throughput, high-dimensional systems biology approaches, which aims to predict differences in protective or suboptimal immune responses to vaccines (12). In this regard, the basis of personalized and predictive vaccinology is the assessment of an individual's genetic background that may impact vaccine immunogenicity and efficacy. Thus, far this approach has been mostly conducted in healthy subjects leading to important findings (13). However, these data can only be partially translated in to specific populations. We recently described distinct transcriptional signatures of purified B and T cell subsets in vertically HIV infected children that was able to distinguish between patients able to respond to Trivalent Inactivated Vaccination (TIV) compared vs. non-responders (14, 15). In addition, purified H1N1 specific B cells showed significant differences in P-TEN/PI3KC2B pathway between responders and non-responders (16, 17).

Following the idea that a single vaccine cannot “fit all” (9), we here aimed at developing a predictive score of poor or protective vaccination immune response to seasonal flu vaccination through an artificial intelligence approach fed by data deriving from a novel *in vitro* gene expression testing approach (IVIGET) in HIV infected patients differentially responding to TIV. We here showed that a multiplexed gene expression analysis from sorted lymphocites subsets in different *in vitro* conditions was able to feed an artificial inteligence model able to select predictive features of influenza vaccination immunogenicity in a pediatric population with suboptimal immune response upon the influenza vaccination.

## Methods

### Study Subjects

Twenty-three subjects vertically infected with HIV-1 (abbreviated as HIV) and on suppressive anti-retroviral therapy (ART) were enrolled between September and November 2012 at Bambino Gesù Children's Hospital, Rome, Italy. Written informed consent was obtained from all subjects or parents/legal guardians upon enrolment and the study was approved by the Institutional review board of the Bambino Gesù Children's Hospital. PBMCs and plasma were isolated by density gradient isolation [46] collected pre (T0) and 21 days post vaccination (T1) and cryopreserved and processed for study at a later date. Serum samples were stored at −80°C.

### Immunization and Sample Collection

Patients were immunized with a single dose of Inactivated Influenza Vaccine Trivalent Types A and B (Split Virion) VAXIGRIP® (sanofi pasteur). The strains for the 2012–2013 season were: A/California/7/2009 (H1N1) pdm09-like strain (abbreviated as H1N1), A/Victoria/361/2011 (H3N2)-like strain (abbreviated as H3N2) and B/Wisconsin/1/2010-like strain (abbreviated as B).

### Hemagglutination Inhibition (HI) Assay

The antibody titers to the H1N1, H3N2 and B influenza strains in sera from HIV and HC were evaluated separately by HI assay (18). The virus strains used in the HI assay were A/California/7/2009 (H1N1) pdm09-like strain, A/Victoria/361/2011 (H3N2)-like strain and B/Wisconsin/1/2010-like strain according to the 2012–2013 influenza vaccine formulation. The HI assay was performed as previously described (18). The HI antibody titers were expressed as the reciprocal of the highest serum dilution at which hemagglutination was prevented. (http://www.gmp-compliance.org/guidemgr/files/021496EN.PDF).

### ELISPot

PBMCs collected at T0 and T1 from HIV and HC were thawed and policlonally activated *in vitro* in complete RPMI medium (Invitrogen) supplemented with 2.5 μg/mL CpG type B (Hycult biotech), 20 ng/mL IL-4 (Peprotech) and 20 ng/mL IL-21 (ProSpec). Cells were harvested after 5 days of culture at 37°C. ELISpot 96-well filtration plates (Millipore) were coated with the addition of purified H1N1, H3N2, and B influenza inactivated virus particles and subsequently loaded with 2 × 10^5^ cells/well. Membranes were punched out with an Eli.Punch device and developed spots were scanned with an Eli.Scan and counted with the ELISpot Analysis Software V5.1 (all from A.EL.VIS).

### Determining Vaccine Response Status

T0 and T1 samples were employed to evaluate patient's ability to respond to the vaccination as previously described (17). Response to vaccinations was determined both by ELISPot for the 3 strains of Flu vaccines (H1N1, H3N2, B) and by Haemagglutination-Inhibition assay (HIA) detected at the time of immunization and 21 days after vaccination as previously described (14, 15). In order to compare patients with differential ability to respond to the vaccination, patients were first selected according to their seroconversion to H1N1 21 days after the immunization resulting in 2 groups, Seroconverter (HIA fold increase ≥ = 4) and Non Seroconverter (HIA fold increase < 4). As additional criteria, patients were selected according to the ELISpot responses for H1N1 at 21 days after immunization as ELISpot negative (<80 H1N1 specific spots/106 PBMCs) and ELISpot positive (>80 H1N1 specific spots/106 PBMCs). According to these 2 criteria we could select among the HIV infected children 12 non responders (NR; HIA fold increase <4 AND H1N1 specific spots <80/106 PBMCs), 11 responders (R; HIA fold increase >= 4 AND H1N1 specific spots >80/106 PBMCs).

### *In vitro* Stimulation, Cell Sorting, and RNA Extraction

T0 PBMC were thawed and cells were counted with Countess Automated Cell counter (Life technology). Cells were resuspended in complete RPMI medium at a concentration of 5 × 10^6^ PBMCs/mL and left at 37°C for 16 h in the presence or absence of H1N1 A/California /09 HA peptides in a final concentration 20 μL/mL. PMBCs were stained for surface markers, Vivid (Pacific Blue), CD10 (PECy7), CD20 (PE), CD27 (APC), IgD (FITC), CD21 (PECy5) for the B cell panel for 15 min and for CD3 (AmCyan), CD4 (PerCP Cy5.5), CD45RO (ECD), CCR7 (Alexa Fluor 700), and CXCR5 (Alexa Fluor 647) and a live/dead marker (ViViD; Molecular Probes) for the T cell panel for 15 min. Subsequently, stained PBMCs were washed twice in PBS, finally filtered with a 40 uM mesh and sorted by FACSAriaII (BD Biosciences). The purity of the sorted cell populations were typically >99%. All antibodies were previously titrated. Viable lymphocytes were identified as live dead amine dye negative (ViViD-) cells (Invitrogen).

**Figure 1:**
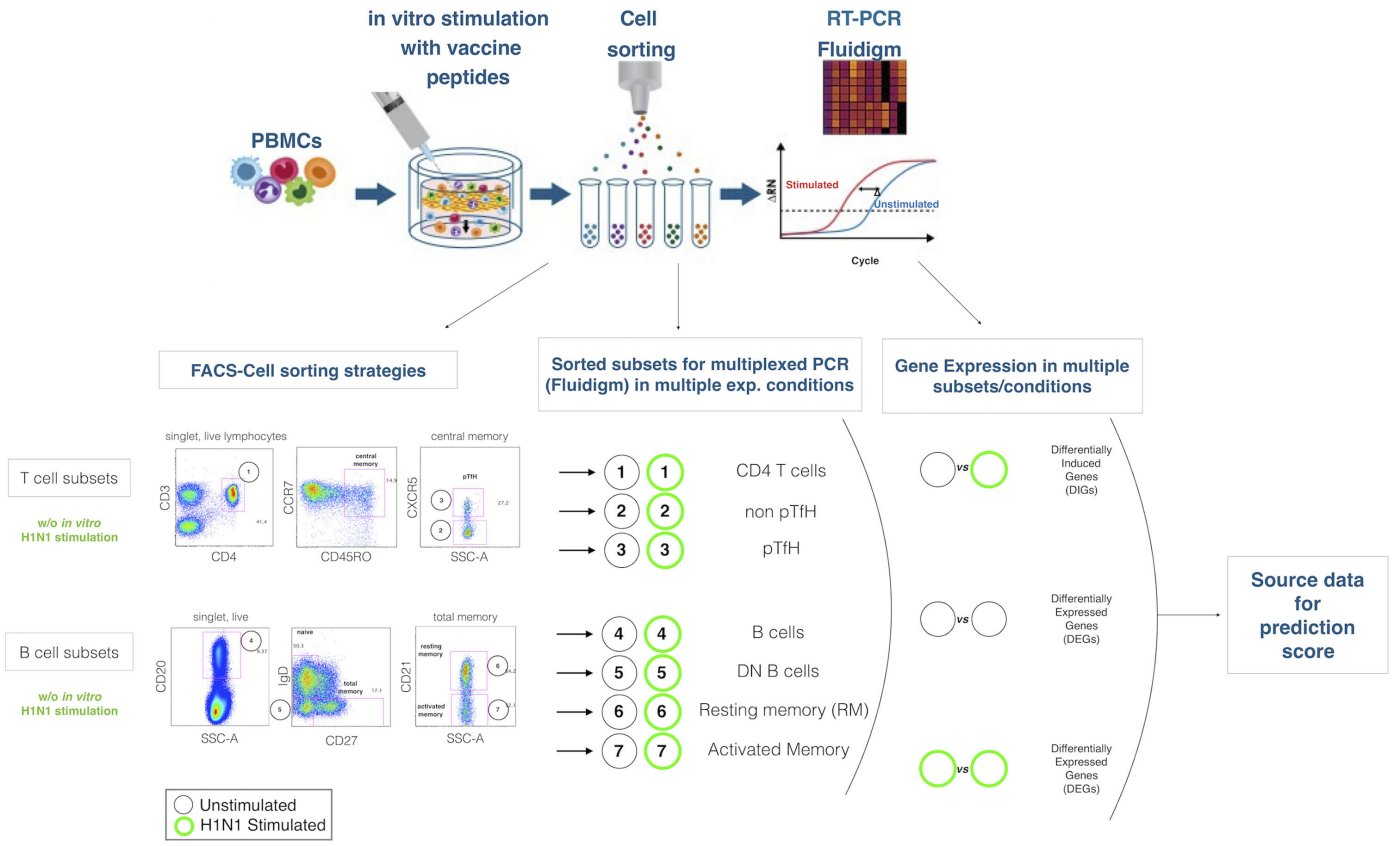
Experimental design. The cartoon on the top panel depicts the experimental procedure. Briefly, total PBMCs are in 2 aliquotes, one stimulated and the latter unstimulated. After sorting lymphocite subsets, gene expression is analyzed by Fluidigm Biomark. Bottom panel describes the gating strategies and the lymphocites subsets selected for sorting and gene expression analysis. Mathematical analysis applied on the subsets in order to obtain differentially expressed genes (DEGs) and differentially induced genes (DIGs) are described.

Five-Hundred live cells per B and T cell subset were sorted into tubes previously loaded with 9 μL of PCR buffer (see also Figure 1 for gating strategy). After sorting, cells were immediately centrifuged (3000RPM for 3 min) and kept on ice. Samples were subsequently transferred in PCR tubes and 18 PCR cycles were performed on a C1000 Thermal Cycler (Bio Rad) with the following scheme (50°C for 20′, 95°C for 2′, 95°C 15″, 60°C for 4′. Last step repeated 18 times). Cells were finally kept at −20 until further analysis. PCR buffer premix for cell sorting contained the following: Cells Direct Reaction mix 5 μL, DEPC water 1,4, Superscript III + Taq 1 μl, 0.2x diluted assay (96 primer mix) 2.5 μL, Superasein 0,1 μL.

### Multiplexed RT-PCR

Previously amplified samples were loaded on a Fluidigm 96.96 standard chip following manufacturer's instructions. Briefly, assay pre-mix was prepared 1:1 20X TaqMan Gene Expression Assay (Applied Biosystems) and Assay Loading Reagent (Fluidigm, Biomark®). The sample pre-mix was prepared with TaqMan Universal PCR Master Mix (2X)(Applied Biosystems), 20XGE Sample Loading Reagent (Fluidigm), and cDNA. Full list of the two panels of gene probes (B subsets and T subsets is shown in Supplemental Table 1 and 2). 5 μl of Assay and Sample mix were loaded into the chip according to manufacturers instructions. Genes' selection has been made according to previous analysis on RNA Sequencing on HIV infected children from a different cohort (data not shown), the literature and online gene banks and biological queries.

Cycle threshold value (Ct) deriving from exported files was corrected according to number of cells sorted if lower than 500. Calculations were made following the expression 67, 5/500 = Y/X where X is the number of cells sorted and Y is the cells equivalent cDNA of cell sorted. The dilution factor (n) was calculated as *n* = 67, 5/Y, and base 2 log of n was subsequently subtracted to Ct value in order to get Corrected Cycle Threshold (c-Ct). Expression threshold (Et), which was used for the main analysis was finally obtained with 40-cCT. Once exported and corrected, data were analyzed through Fluidigm SingluaR (SingulaR analysis toolset 3.0) package, loaded on R (software R 3.0.2 GUI 1.62). As previously described (De Armas, 2017) gene expression differences between different groups within same subset and condition were used to identify Differentially Expressed Genes (DEGs). Alternatively, paired gene expression differences between stimulated (stim) and unstimulated samples (med) (stim-med) within the same subset were used to define Differentially Induced Genes (DIGs). All raw data on gene expression analysis used for the present project are available on the Gene Expression Omnibus: NCBI gene expression and hybridization array data repository (GEO) (GSE155730).

### Bioinformatics and Statistical Analysis

#### Predictive Modeling Framework

**Figure 2:**
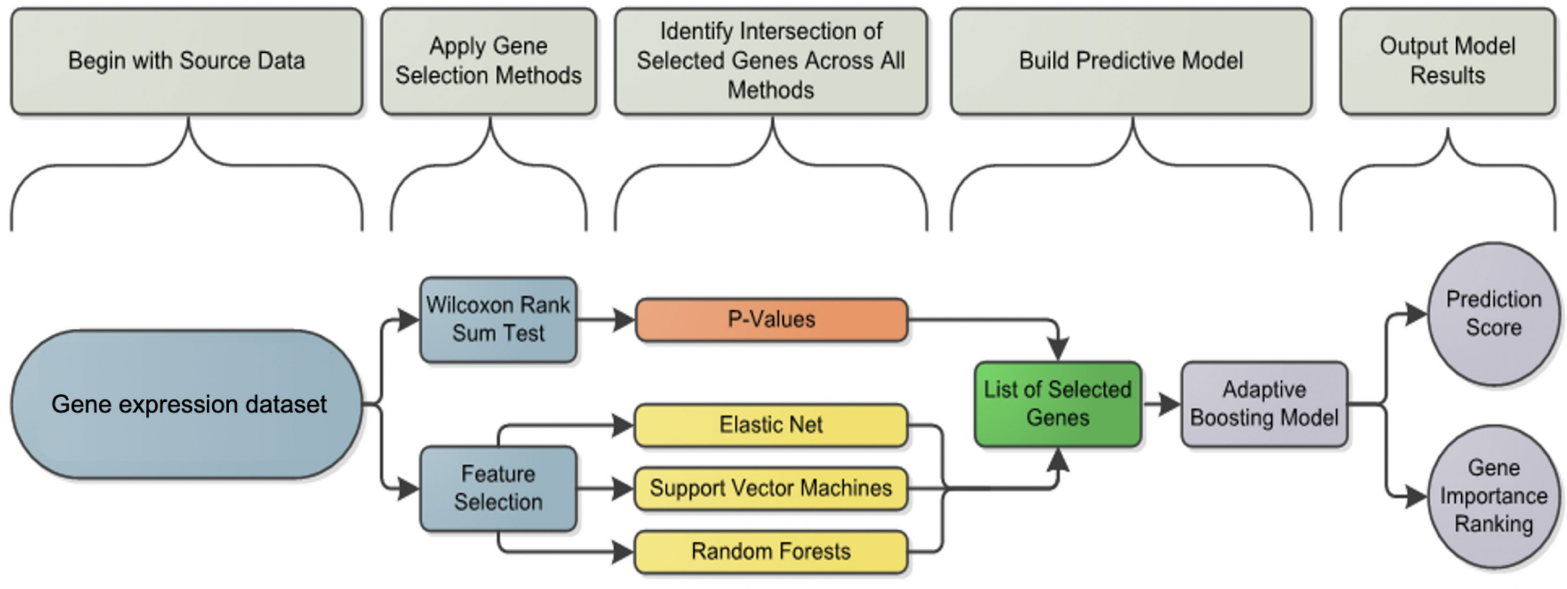
Analysis framework flowchart: pipeline workflow for predicting the vaccine response. Differentially induced genes between responders and non-responders were selected using a machine learning feature selection based on three different algorithms and the Wilcoxon test for each cell subset and condition. The list of selected genes was used by the Adaptive Boosting algorithm to build the predictive model and calculate the prediction score.

We propose a workflow (Figure 2) used for gene selection and model building that use the 96 genes with age and sex as covariates. This method was applied to each subset of B cells (AM, DN, REM) and T cells (CD4, NT, PBMC, TFH), further divided into two conditions, namely stimulated (stim), unstimulated (med) and the derived data of the stim-med, for 23 patients. In addition, this workflow has also been applied to the entire dataset (B and T cells) to obtain a predicted probability score. Due to the small sample size and high dimensional data, the Wilcoxon Rank Sum Test was used to select genes whose expression levels are different between responders and non-responders. As compared to other feature selection methods, this test outperforms others in terms of accuracy and robustness (19) Using the two-sided test to evaluate whether these two subpopulations had different gene levels, *p*-values were derived to assess significance at α = 0.05. Genes with significantly different expression levels were used in the next step of the analysis framework. The feature selection process was applied to each dataset to select those genes that are predictive of response to vaccine. Applying multiple machine learning methods, each using a different approach, increased the confidence in selecting the best genes for the model.

The machine learning methods used were Elastic Net (glmnet function in R) (20), Support Vector Machines (svm.fs function in R) (21) and Random Forests (randomForest function in R) using 3-fold cross validation repeated 8 times (22). Variable importance was also calculated by Random Forests and used to define the gene importance ranking as previously described (22). If a gene or feature was selected at least 10 times in total throughout the process, then it was considered for further analysis in the prediction model.

**Figure 3:**
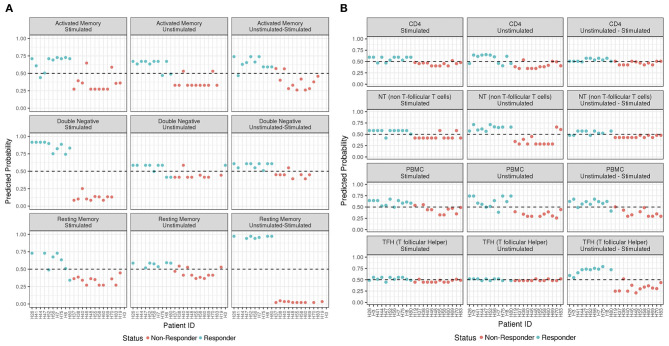
ADA Boost Probability Scores for T Cells **(A)** and B cell subsets **(B)**. The probabilities of prediction are shown for each patient (the non-responder in red and responder in blue). If the probability is >0.50 the patient has been classified as responder, on the contrary if <0.50.

**Figure 4:**
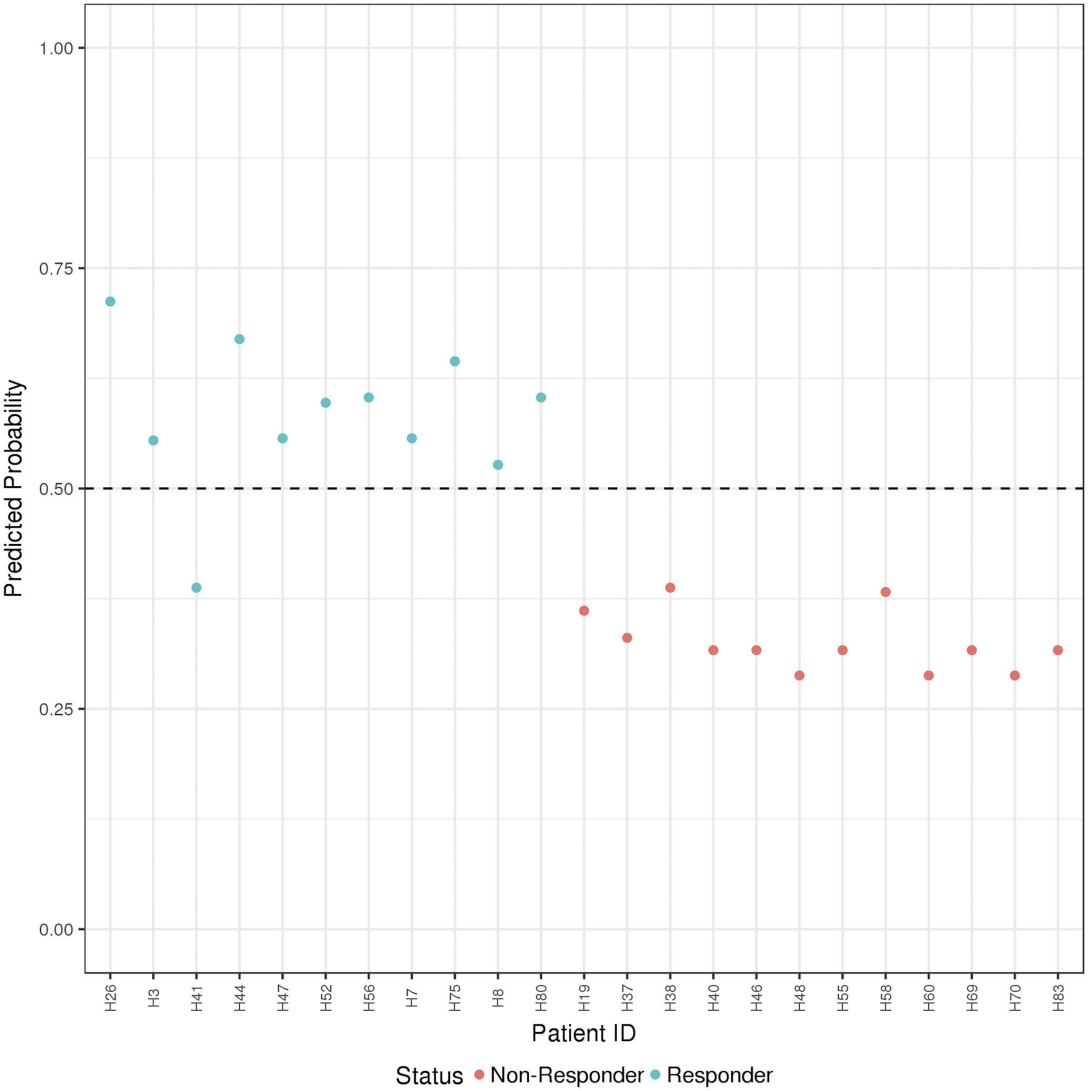
ADA Boost Probability Scores (B and T Cells): the combined prediction score between B and T cells are shown for each patient (the non-responder in red and responder in blue). If the probability is >0.50 the patient has been classified as responder, on the contrary if <0.50.

After selecting a subset of features by using the Wilcoxon Rank Sum Test and machine learning, an Adaptive Boosting model using continuous predictors and generating predicted probabilities of response was used as the final predictive model. The Adaptive Boosting model (http://rob.schapire.net/papers/explaining-adaboost.pdf) was used as it is less susceptible to overfitting and attempts to combine rules to create a more accurate prediction. This model was implemented in R using the caret (http://topepo.github.io/caret/index.html) package. The ADA Boost method uses a training set, a subset of the data that is set aside, and assigns a ±1 as classifier values. A classifier value indicates how important a feature is for the model. The classifiers are then weighted based on the training set, and the prediction is recalculated. Using the data, the program will assign weights to features beyond the ±1 classifier at every stage. To obtain the final results of the model, ADA Boost uses the sum of every weight and classifier combination to provide a probability of response. The result of the model is a predicted probability of response for each patient (Figures 3, 4). Considering both the Wilcoxon and feature selection significant genes, the final model uses the intersection (B cells) or union (T cells) of the genes across subsets. The R statistical software version 3.0.3 was used for all analyses (www.r-project.org).

The R package “enrichR” v2.1 was used to perform functional annotation and pathway enrichment analysis on the genes selected to build the five models with the best prediction precision.

## Results

### Patients' Characteristics and H1N1 Response to Influenza Vaccination

To define the ability to elicit memory response upon H1N1 of the trivalent inactivated Influenza vaccination 2012/2013 (TIV) we investigated hemoagglutination inhibition assay in 65 HIV infected children under stable and highly active antiretroviral treatment (HAART) and viral control at the time of vaccination. Clinical characteristics are listed in Table 1. Study participants were classified as vaccine responders (R) and vaccine non-responders (NR) according to the criteria established by Food and Drug Administration Guidance for Industry as previously described (14). R were characterized by HAI titer to H1N1 antigen (Ag) at T1 of >1:40 and > four-fold increase compared to baseline. In order to validate our criteria of selection, even considering the lower reliability of serological correlates in such patients, we applied an additional measure of vaccine responsiveness in our study population performing the B cell ELISpot response to H1N1 Ag (≥ or < 80 spots /106 PBMCs in responders (R) and non-responders (NR), respectively).

**Table 1:** Study subjects' characteristics.

**Baseline characteristics**	**HIV NR**	**HIV R**
Age years, mean (SEM)	15.16 (2.1)	13.72 (2.3)
n (female)	12 (7)	11 (5)
%CD4+ T cells, mean (SEM)	37.97 (4.9)	32.49 (6.0)
HIV RNA <50cp/mL, n	11	10
IgG (mg/dL) (mean)	1387.4	1,356
IgM (mg/dL) (mean)	135.1	118.9
IgA (mg/dL) (mean)	210.7	225.1
CDC (A/B/C) (1/2/3)	(3/4/5) (3/4/5)	(2/5/4) (4/3/4)
Lymphocites/mm^3^ mean (SEM)	2494 (278,9)	3109 (363,1)
WBC 10^3^/uL, mean (SEM)	7.6 (1.5)	7.3 (0.7)
ART regimen (2 NRTI+PI-r/2 nNRTI+ NRTI/2 NRTI+ii)	(5/5/2)	(5/4/2)

*SEM, standard error of the mean; CRP, C-reactive protein; CDC, Center for Disease Control classification of AIDS. WBC, white blood cells. ART, antiretroviral treatment; NRTI, Nucleoside and Nucleotide Analog Reverse Transcriptase Inhibitors; PI, Protease Inhibitors; nNRTI, Non-Nucleoside Analog Reverse Transcriptase Inhibitors; ii, Integrase Inhibitors*.

### Features Selection and Identification of Predictive Score Through Artificial Intelligence

In order to predict the vaccination response based on the expression levels of 96 genes, we have implemented a bioinformatic pipeline that was tested on six sorted subsets of B and T cells, as reported in Figure 1 and PBMC in 23 patients. For each subset, the conditions of stimulation (stim), non-stimulation (med) and the difference between the two (stim-med) have been considered. For each of these subset/condition, the algorithm, reported in Figure 2, selects the Differentially Induced Genes (DIGs) and Differentially Expressed Genes (DEGs) whose expression levels can, better than others, discriminate responding individuals from non-responders upon TIV. A total of 179 genes/conditions were initially selected among the different subsets and conditions (Table 2). As shown in Table 2, a specific number of genes were respectively, selected for the med (55 genes), stim (62 genes), and med-stim conditions (62 genes). Subsequently, these genes were then used to build statistical models. The ADA Boost models generated for each category returned a probability score that estimates the classification of each patient in responder (R) and non-responder (NR). Assuming a predicted probability >0.50 classified as a responder, the ADA Boost model was able to predict R and NR in specific subsets and conditions according to the previously selected genes. Indeed, the Resting Memory (REM) med-stim, Double Negative (DN) stim, TFH med-stim and PBMC med datasets showed the best results in terms of predicted probability as shown in Figure 3. No mispredictions were found in REM med-stim, DN stim and PBMC unstim, whereas only one misprediction was found according the ADA boost of TFH med-stim.

**Table 2:** Selected genes and conditions.

		**Condition**		
**Cell type**	**Cell subset**	**Med**	**stim**	**Med-stim**
B	AM	1	2	5
	DN	5	9	5
	REM	8	8	3
T	CD4	7	9	12
	NT	12	14	12
	PBMC	18	9	12
	TFH	4	11	13
Total		55	62	62

In order to provide a comprehensive description of gene expression with higher accuracy in terms of prediction probability, all gene expression analyses, from multiple subsets and conditions were ranked.

Table 3 summarizes the classification accuracy and a relative ranking for each category. Rankings were used to identify the cell subsets and conditions that yielded high prediction accuracy as well as a wider range of predicted probability values. Correct prediction ranged from 68% up to 100% when tested on the cohort. Following these criteria, five cell subsets/conditions providing the highest classification accuracy combined with the highest predicted probability were highlighted (Table 3).

**Table 3:** Subsets and conditions importance ranking.

	**Correct classification**			**Predicted probability of response**			
**Cell type, subset, Condition**	**No. Patients**	**Correct (%)**	**Rank**	**Minimum**	**Maximum**	**Range**	**Rank**
B_REM_med_stim	17	100%	2	0.022	0.972	0.950	1
B_DN_stim	20	100%	1	0.084	0.916	0.832	2
T_TFH_med_stim	22	95%	3	0.210	0.790	0.580	3
T_PBMC_med	21	90%	6	0.258	0.742	0.484	4
B_AM_med_stim	21	86%	12	0.261	0.739	0.479	5
B_REM_stim	19	89%	7	0.271	0.729	0.459	6
B_AM_stim	22	86%	10	0.273	0.727	0.453	7
T_NT_med	22	91%	5	0.286	0.714	0.429	8
T_PBMC_med_stim	21	86%	13	0.296	0.673	0.377	9
T_PBMC_stim	21	86%	14	0.326	0.674	0.347	10
B_AM_med	21	81%	16	0.329	0.671	0.343	11
T_CD4_med	23	74%	20	0.347	0.653	0.305	12
B_REM_med	18	83%	15	0.366	0.593	0.227	13
B_DN_med_stim	17	94%	4	0.392	0.608	0.217	14
T_CD4_stim	23	87%	8	0.404	0.596	0.192	15
B_DN_med	19	79%	17	0.414	0.586	0.172	16
T_NT_stim	23	87%	9	0.418	0.582	0.164	17
T_CD4_med_stim	23	78%	18	0.427	0.573	0.146	18
T_NT_med_stim	22	86%	11	0.429	0.571	0.142	19
T_TFH_stim	23	78%	19	0.447	0.553	0.106	20
T_TFH_med	22	68%	21	0.481	0.519	0.038	21

*All subsets and conditions were ranked both for the accuracy of classification and for the expected probability range. According to both rankings, the categories with the best classification capacity, highlighted in red, were selected for the final score*.

Finally, the B and T cells subsets/conditions were used to calculate a combined score. The score, was then tested in our cohort of patients which were blindly predicted as responders and non-responders. In this case, as shown in Figure 4 and Table 4, only one patient out of the entire cohort was misclassified providing a prediction accuracy of 95.6%.

**Table 4:** Cross validation of the model.

**ID**	**Non responder**	**Responder**	**Predicted**	**Observed**
H19	0.639	0.361	Non.Responder	Non.Responder
H26	0.288	0.712	Responder	Responder
H3	0.445	0.555	Responder	Responder
H37	0.669	0.331	Non.Responder	Non.Responder
H38	0.613	0.387	Non.Responder	Non.Responder
H40	0.683	0.317	Non.Responder	Non.Responder
H41	0.613	0.387	Non.Responder	Responder
H44	0.331	0.669	Responder	Responder
H46	0.683	0.317	Non.Responder	Non.Responder
H47	0.443	0.557	Responder	Responder
H48	0.712	0.288	Non.Responder	Non.Responder
H52	0.403	0.597	Responder	Responder
H55	0.683	0.317	Non.Responder	Non.Responder
H56	0.397	0.603	Responder	Responder
H58	0.617	0.383	Non.Responder	Non.Responder
H60	0.712	0.288	Non.Responder	Non.Responder
H69	0.683	0.317	Non.Responder	Non.Responder
H7	0.443	0.557	Responder	Responder
H70	0.712	0.288	Non.Responder	Non.Responder
H75	0.356	0.644	Responder	Responder
H8	0.473	0.527	Responder	Responder
H80	0.397	0.603	Responder	Responder
H83	0.683	0.317	Non.Responder	Non.Responder

*Predicted and observed outcome are listed for all patients*.

Due to the small sample size, in order to overcome the unfeasibility to perform a nested cross valitation, we confirmed the stability of the accuracy in features' selection of the top 5 B/T subsets/conditions resampling the dataset according to these 5 subset/condition. This re-analysis confirmed the stability in feature's selection of the initial model. Indeed, all subset models out of the 5 selected cell subsets/ condition feature displayed between 80 and 100% accuracy according to the bootstrap replications (Supplementary Table 3). The best performing subset by this metric was the B DN med_stim subset with a confidence interval ranging from 94 to 100%. All genes from the subset models had 50–90% selection rates in bootstrap replicates.

### Functional Analysis of Genes Selected by Artificial Intelligence

**Figure 5:**
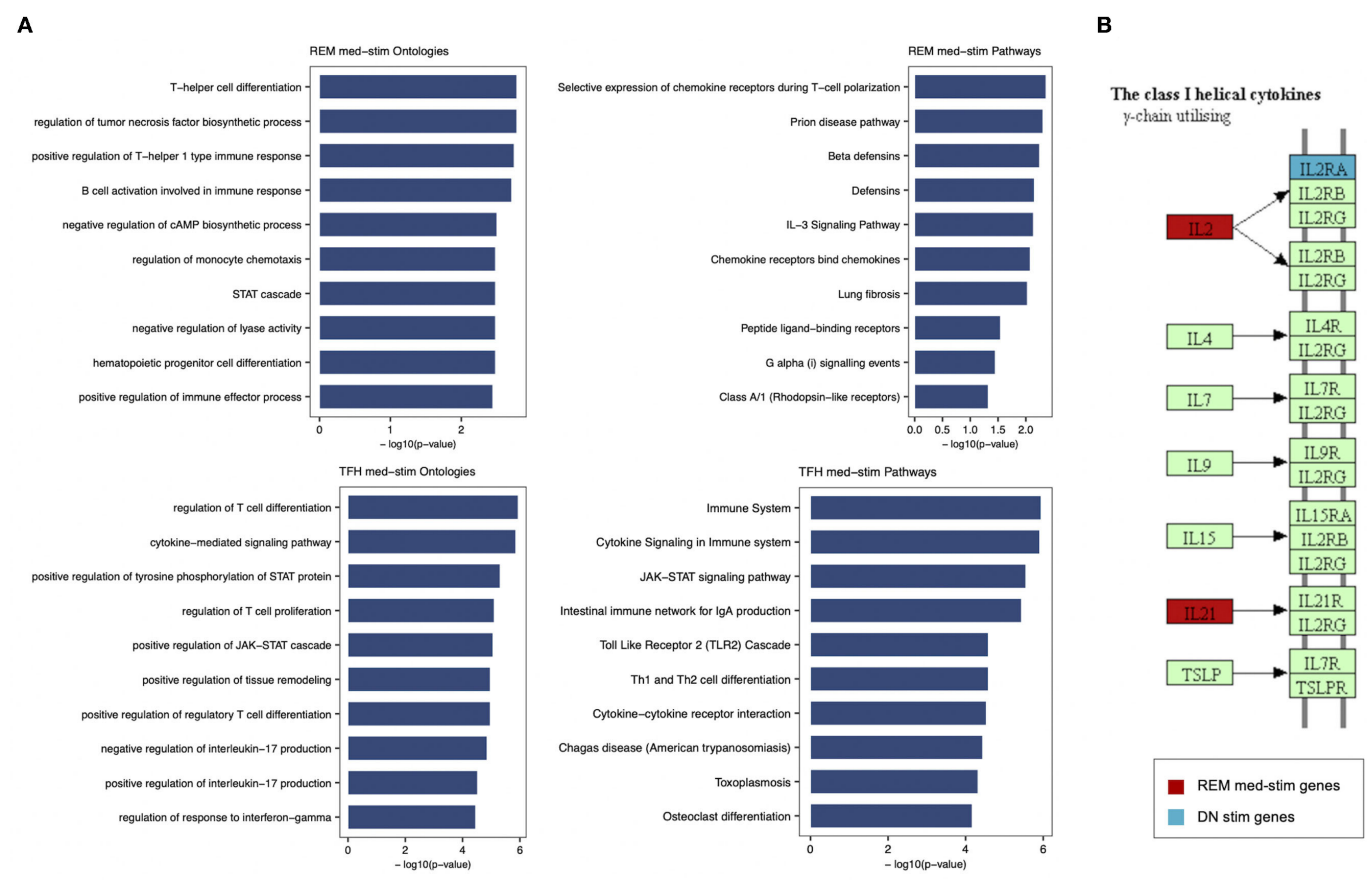
Enrichment analysis performed for REM med-stim and TFH med-stim genes on GO terms and pathways. **(A)** Bar plots with the top 10 terms sorted by *p*-value. **(B)** Cytokine-cytokine receptor Kegg map that shows in red the REM med-stim genes and in blue the DN stim gene.

In order to characterize the biological functions of the genes used to build the five models with the highest prediction accuracy, we performed a functional enrichment analysis on the five sets of genes shown in Table 5. According to gene set enrichment analysis, the REM med_stim selected genes associated with transcription pathways of chemokine expression and T cell-oriented proliferation (Figure 5). These results are in line with ontologies which were particularly enriched in the positive regulation of T helper I type immune responses (Figure 5). In the T cell counterpart, pTFH cells, several genes involved in cytokine-cytokine mediated signaling were enriched. Also JAK-STAT signaling and TLR oriented stimulation pathways were upregulated (Figure 5) in patients able to respond to TIV. It is important to mention that other genes, such as *IL21* and *TNSF13* (APRIL), previously reported to be crucial in the T-B cell interaction (23, 24), resulted informative after *in vitro* stimulation to define responders. According to our previous analysis (15) these data may suggest that the functional expression of these genes after *in vitro* stimulation is able to predict the ability of these cells to activate a functional cascade which sustain an effective humoral response after vaccination.

**Table 5:** List of the genes used to build the five models with the highest prediction accuracy.

**Subset/condition**	**Gene name**
REM med_stim	BATF, CCR2, CD69
DN stim	DUSP4, HAVCR2, IL2RA, PDL1, PPP3CA, SAMHD1, SELPLG, STAT3, TLR9
TFH med_stim	ABCB1, DUSP4, FOXP3, ICOS, IFNG, IL2, IL21, LAG3, MAPK3, PDCD1, PDL1, SOCS1, TNFSF13
PBMC med	ADAM17, BCL6, CAV1, CCR6, GATA3, IL6RA, IL6ST, PKC.A, BST2, CD3D, CXCR4, ICOS,, ID2, IFNG, IL21R, IRF4, MAF, PTX3
DN med_stim	CAMK4, MX1, SELPLG, SOCS1, TLR9

*In red are shown the up-regulated genes for the med and stim categories (DN stim, PBMC med) and the genes with the highest Δ (stim-med) value in the Responders for the med-stim categories (REM med-stim, TFH med- stim, DN med-stim. In blue are shown the down-regulated genes for the med and stim categories and the genes with the lowest Δ (stim-med) in the Responders for the med-stim categories (REM med-stim, TFH med-stim, DN med-stim*.

Gene ontology analysis also revealed the expression of chemokine receptors with complementary activity between IgD- CD27- Double Negative (DN) B cells and pTFH. Importantly *IL2* and *IL2RA* were both selected in the pTFH and DN, respectively. These data may suggest how activation of this pathway after *in vitro* stimulation represents a functional correlate of plasma cell lineage commitment after *in vitro* stimulation as previously reported in mice (25).

## Discussion

Definitive and predictive biomarkers of vaccination efficacy are still largely unknown and may provide crucial information in the design or improvement of existing vaccines. This gap further applies to specific groups of patients presenting with underlying immunological conditions which increase their risk of suboptimal responses to vaccinations (4, 5). In the present study we developed a predictive score of immunogenicity to seasonal flu vaccination through an artificial intelligence approach fed by data deriving from a novel *in vitro* gene expression testing approach (IVIGET) performed prior to the immunization in a cohort of HIV infected patients.

Systems biology has helped to develop specific predictive assays in the oncology field. Also, targeted molecular assays have played an increasingly important role in identifying prognostic outcomes or predicting response to chemotherapy, starting from tumor biopsies (26). Indeed, these assays, which are now routinely performed in local pathology labs to help guide treatment decisions in breast cancer (27) lung cancer (28), and colorectal cancer (29), have been tested and validated on tumor biopsies.

On the other hand, systems vaccinology has been often analyzed in total blood or cell suspensions (e.g., PMBCs) which present an high intrinsic variability due to transitory confounding effects (e.g., concomitant infections or vaccination, inflammation, systemic immune deficiency, etc.) which may represent important variables making the aim of systems biology even more challenging. In addition, specific changes in cell frequency due to underlying immune defects or to physiologic conditions (i.e., age, pregnancy) may importantly interfere with the analysis of functional correlates of vaccine efficacy (11, 30).

Additional confounding effects are represented by inter-individual differences such as gender, age, pre-existing immunity, microbiota or systemic conditions which may further affect data analysis and their interpretation (31–33).

Following this idea, over the last few years we have described trancriptional signatures of vaccine response from purified lymphocyte subsets or single Ag specific cells in VPs (14, 16, 17). Our data demonstrated how the analysis of purified cell subpopulations may provide additional information compared to total PBMCs, and how gene expression analysis after *in vitro* stimulation may provide distinct predictive correlates of Ab and cellular response upon TIV (16) in VPs. In the present study, our analysis approach complement the evidence produced on single subsets applying state-of- the-art machine learning and methodology to the *in vitro* gene expression testing (IVIGET) which is focused on cell subsets directly involved in the immune responses upon Influenza vaccination. After multiple gene selection methods were applied for all subsets and conditions the score was interrogated at the time of vaccination on the ability to predict immune response to TIV in a previously investigated cohort of HIV infected patients (14). Albeit limited by the small sample size, which made the nested cross validation unfeasible, our analysis was able to perform a selection of genes and conditions able to predict vaccination response in specific B and T cell subsets. Conditions with higher prediction probability and correct classification were further ranked and selected to produce the final score which was blindly tested on the cohort. To further overcome the contamination of the test set, the 5 top ranked subsets after single-model re-analysis confirmed the stability of the accuracy and suggests how the model is able to build a predictive score for vaccination response by selecting important subset/condition to be validated in larger scale studies. Four out of five of the subset/condition selected for the final score included the stimulated condition and more precisely three out of the five refer to data derived from the difference in gene expression between the stimulated and unstimulated condition. Overall these results suggest that this *in vitro* stimulation approach in combination with others *in vitro* tests recently described (34) may provide important information in term of prediction of vaccine responsiveness and early pre-clinical selection of effective vaccine candidates for VPs. Our data may thus confirm that gene expression after a relatively short (16 h) *in vitro* stimulation may emulate early transcriptional changes that were analyzed *in vivo* both in mice (35) and in humans (36, 37). Early transcriptional changes, derived from whole transcriptome sequencing from blood samples collected at day 0, 1, 3, and 7 after immunization were shown to be informative in predicting long-term humoral and cell mediated responses to Hepatitis B, Ebola (38) and yellow fever (36) vaccinations. Interestingly the majority of differentially expressed genes (DEGs) resulted from the analyis between day 1 or day 3 and day 0 suggesting that early signatures were able to orchestrate and correlate with long term memory responses. In line with this, our analysis revealed how the majority of selected features were among Differentially Induced Genes (DIGs) after a peptide mix stimulation of 16 h). Following these evidence we also recently reported how early signatures after *in vitro* stimulation in Ag specific B cells were able to define the B cell fate after re-encountering of the antigen (39). Overall these findings suggest that both the analysis of purified cells, directly involved in the immune response triggered by a peptide-specific stimulation may provide distinct signatures of immunogenicity that may be useful to implement vaccination predictive tools.

It is important to consider as a limit of the tools presented here, that effectiveness of the score may be specific to the seasonal influenza vaccination (e.g., 2009) and may not apply to other viral strains that make up the vaccine as it continuously changes over the years. It was noted by Nakaya et al. that transcriptional differences differed between the Live Attenuated Influenza Vaccine and the TIV with respect to both classes of genes and cell subsets orchestrating the early immune response (40). Indeed, in a targeted microarray confirmatory analysis on sorted subsets, B cells showed higher DEGs in TIV vaccinee compared to LAIV with a peculiar enrichment in Antibody secreting cells genes (39, 40). Additional studies will be needed to cross validate the score in yearly vaccinated patients and in a vaccine-type/year specific manner. The overfitting caused by the model may represent another limit of the study (41). To reduce this potential problem, we adopted the Adaptive Boosting algorithm which is less susceptible to overfitting through implicit regularization and attempts to combine rules to create a more accurate prediction (http://rob.schapire.net/papers/explaining-adaboost.pdf). Moreover, we have implemented a robust feature selection based on four different methods and we used a dataset balanced between responders and non-responders. To further reduce the risk of overfitting and to increase the accuracy of the models, it would be necessary to increase the sample size and possibly use two independent datasets for the testing and training phases (42).

As previously discussed, the score applies on a relatively limited and curated panel of genes that cannot provide a complete mechanistic insight on the biology orchestrating the immune response upon TIV. However, the genes selected by the score confirm the accumulating evidence on B and T lymphocytes functional data which have been produced in the last few years in patients differentially responding to TIV. Indeed, previous results in pTFH after *in vitro* stimulation highlight the importance of IL21, found upregulated in R (43), confirming previous report in children, adults and elderly able to respond to TIV (44). Also the IL2 pathway, in line with previous evidence (42, 43), seems negatively correlated to the ability to respond to the vaccination when over expressed by pTFH. Overall, these data suggest how IL2 expression triggers a Th1 oriented immune response, rather than long term memory, which was confirmed in another study investigating the correlation between circulating TfH and immunogenicity upon Ebola virus vaccination (44). Our data further add information about the IL2 pathway in the B cell compartment as it was noted that IL2RA was included by the gene selection of DN after *in vitro* stimulation. The IL2 effect on human naïve B cells was recently investigated for the ability to induce plasmacell differentiation through ERK signaling after BACH2 silencing (25). For the first time, we showed that in TIV non responders, IL2RA receptor was upregulated in the so called “double negative” B-cell subset (expressing neither CD27 nor IgD), recently reported to be accumulated in aging populations (45). Overall, these data may suggest how the lack of downregulation in the B cell counterpart after IL2 production from the circulating TFH may interfere with an adequate memory response. Additional studies on this subset will be needed in order to define whether a manipulation or an adjuvanted vaccination specifically targeting the IL21/IL2 molecule production and receptors expression may increase vaccine immunogenicity.

Also Resting memory after *in vitro* stimulation were selected by the score as an informative subset of TIV response. BATF, a transcription factor which was recently showed to induce plasmacell differentiation of memory B cells after CD40L/CD40 signaling (46) was upregulated after H1N1 peptides *in vitro* stimulation of TIV responders. Also CD69 and CCR2 genes emerging from the score in the Resting memory subset confirm the importance of a T cell mediated response.

Although this analysis cannot provide a full mechanistic insight of the molecular mechanisms underlying the immune response since it is performed on a curated and limited panel of genes rather than the full transcriptome, it is promising in providing functional correlates to be used in a prediction score. Additional mechanistic analysis with deeper transcriptional analysis should confirm findings from these data.

In conclusion our analysis suggest that the *in vitro* stimulation and gene expression analysis on purified cell subsets that are involved in the immune responses upon vaccination, may represent valuable information to build a predictive score of immunogenicity. These analyses should be supported by future studies with larger sample size in order to validate this score in HIV infected children. These results may inform novel and more effective immunization strategies in HIV infected children and in other vulnerable population presenting with suboptimal immune responses.

Future studies, beyond the current approach, to evaluate protective immune responses remains an important goal to facilitate the interpretation of response to existing and emerging vaccines, particularly in VPs.

## Data Availability Statement

The original contributions presented in the study are included in the article/Supplementary Materials, further inquiries can be directed to the corresponding author/s.

## Ethics Statement

The studies involving human participants were reviewed and approved by Coordinamento Amminstrativo Studi Clinici e Comitato Etico Presidenza IRCCS Ospedale Pediatrico Bambino Gesù. Written informed consent to participate in this study was provided by the participants' legal guardian/next of kin.

## Author Contributions

NC, LD, PP, and SaP conceived the study and designed the experiments. NC and LD performed the experimental procedures. NC and GP drafted the first version of the article. CB, MF, and NC performed statistical analysis and bioinformatics. VS, EM and PZ provided samples and participated to the design of the study. Supervision and resources were provided by PR, PP, and SP. All authors participated in writing, review and editing of the article.

## Conflict of Interest

MF, and CB were employed by BioStat Solutions, Inc. The remaining authors declare that the research was conducted in the absence of any commercial or financial relationships that could be construed as a potential conflict of interest.

## Supplementary Material

Supplementary Figure 1 ELISpot analysis. **(A)** Shows representative plates for ELIspot. Dot plot in panel B shows H1N1 specific B cells per million PBMCs from samples collected 21 days after TIV.

Supplementary Table 1 Gene panels with probe list for B cells.

Supplementary Table 2 Gene panels with probe list for T cells.

Supplementary Table 3 Single model re-analysis on top 5 ranked subsets/conditions.
