# Supplementary figures and images for: Artificial Intelligence Applied to in vitro Gene Expression Testing (IVIGET) to Predict Trivalent Inactivated Influenza Vaccine Immunogenicity in HIV Infected Children
Source: Front Immunol. 2020 Oct 5;11:559590. doi: 10.3389/fimmu.2020.559590 (PMC7569088; doi:10.3389/fimmu.2020.559590)

**a**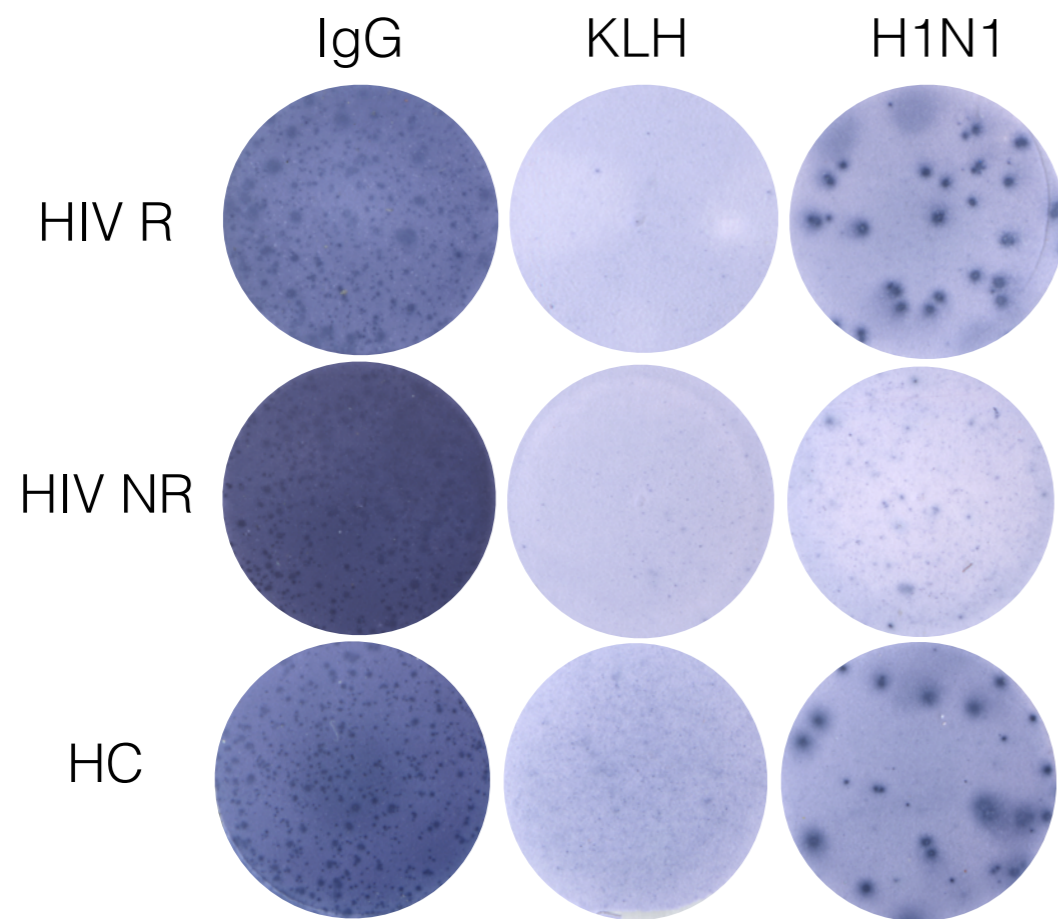**b**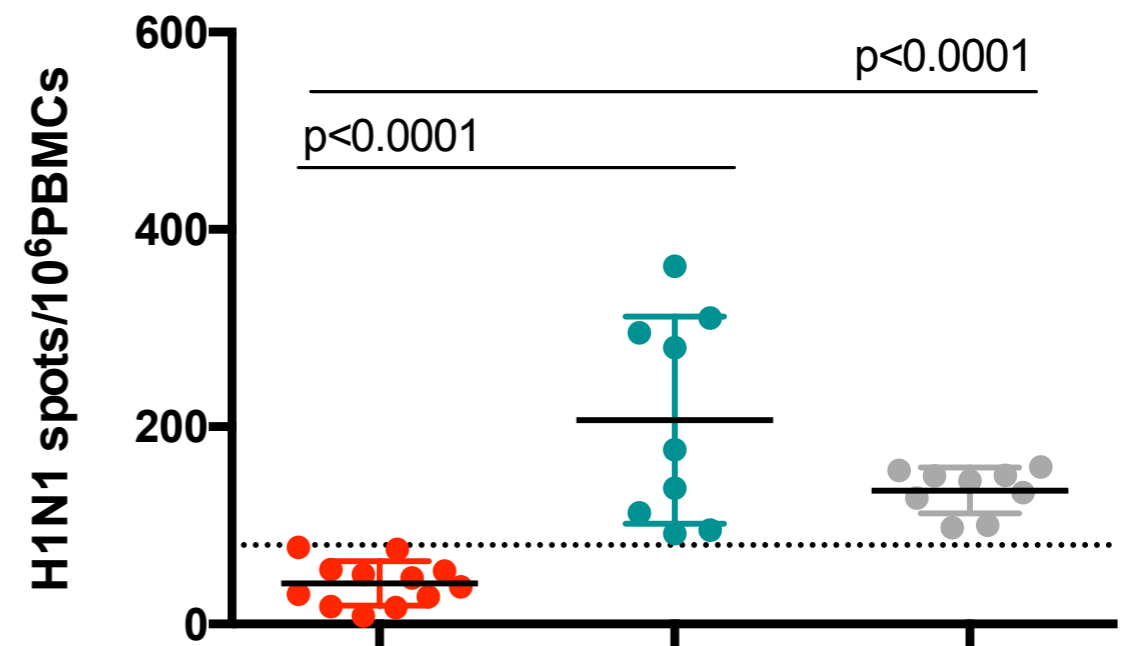

Supplement: Supplementary Figure 1 — ELISpot analysis. (A) Shows representative plates for ELIspot. Dot plot in panel B shows H1N1 specific B cells per million PBMCs from samples collected 21 days after TIV. [file Image_1.pdf]
