# Supplementary material for: Artificial Intelligence Applied to in vitro Gene Expression Testing (IVIGET) to Predict Trivalent Inactivated Influenza Vaccine Immunogenicity in HIV Infected Children
Source: Front Immunol. 2020 Oct 5;11:559590. doi: 10.3389/fimmu.2020.559590 (PMC7569088; doi:10.3389/fimmu.2020.559590)
Supplement: Supplementary Table 1 — Gene panels with probe list for B cells. [file Table_1.DOCX]

Table S1. B cells gene panel for Fluidigm BioMark Experiment

| **Gene name** | **Alias** | **Taqman assay ID** | **Gene name** | **Alias** | **Taqman assay ID** |
| --- | --- | --- | --- | --- | --- |
| BCL6 |  | Hs00277037_m1 | CD27 |  | Hs00154297_m1 |
| CD40L | CD154 | Hs00163934_m1 | CAMK4 |  | Hs00174318_m1 |
| CXCR3 |  | Hs00171041_m1 | CAV1 | BSCL3 | Hs00971716_m1 |
| BCL2 |  | Hs99999018_m1 | NOD2 |  | Hs01550762_g1 |
| IL10 |  | Hs00961622_m1 | MZB1 |  | Hs00414907_m1 |
| SAMHD1 |  | Hs00210019_m1 | TXNDC5 |  | Hs01046709_mH |
| TNFSF13 | APRIL | Hs00182565_m1 | BTK |  | Hs00975865_m1 |
| CD86 | B7-2 | Hs01567025_m1 | IKBKG | NEMO | Hs00415849_m1 |
| ABCB1 | MDR1 | Hs00184500_m1 | CCR7 |  | Hs00171054_m1 |
| CCR2 |  | Hs00356601_m1 | IFNAR2 |  | Hs01022060_m1 |
| CD74 | DHLAG | Hs00959498_g1 | BATF | BCL2L4 | Hs00232390_m1 |
| CXCL10 | IP10 | Hs00171042_m1 | EOMES |  | Hs00172872_m1 |
| LILRB1 | ILT2 | Hs01848117_s1 | NFKB1 |  | Hs00765730_m1 |
| TRIM5 |  | Hs01552559_m1 | SOCS1 |  | Hs00705164_s1 |
| CD79B |  | Hs01058826_g1 | TLR7 |  | Hs00152971_m1 |
| IL10RA |  | Hs00155485_m1 | TLR9 |  | Hs00152973_m1 |
| SELPLG |  | Hs00380945_m1 | BLNK |  | Hs00179459_m1 |
| BST2 | Tetherin | Hs00171632_m1 | IGD | IGHD | Hs00920518_g1 |
| CD38 |  | Hs01120071_m1 | IRAK4 |  | Hs00211610_m1 |
| DUSP4 |  | Hs01027785_m1 | FOXO3 |  | Hs00921424_m1 |
| IFIT2 |  | Hs00533665_m1 | IL2RA | CD25 | Hs00166229_m1 |
| IL6ST | gp130 | Hs00174360_m1 | PDL1 | CD274 | Hs00228839_m1 |
| IRF4 |  | Hs01056533_m1 | PPP3CA |  | Hs00174223_m1 |
| ITCH |  | Hs00395201_m1 | TNFSF13B | BAFF | Hs00198106_m1 |
| PRDM1 | BLIMP1 | Hs00153357_m1 | IGM |  | Hs00941538_g1 |
| PTEN |  | Hs02621230_s1 | IRAK3 |  | Hs00936103_m1 |
| BTLA |  | Rh02889477_m1 | MYD88 |  | Hs01573837_g1 |
| FAS |  | Hs00531110_m1 | NKRF |  | Hs00213907_m1 |
| GATA3 |  | Hs00231122_m1 | PILRB |  | Hs00273801_m1 |
| IL6 |  | Hs00985639_m1 | PPPIR13B |  | Hs00367408_m1 |
| IL6RA |  | Hs00169842_m1 | TIRAP |  | Hs00364644_m1 |
| STAT3 |  | Hs01047580_m1 | TNFRSF13C | BAFFR | Hs00606874_g1 |
| STAT5A |  | Rh02844611_m1 | CYBB |  | Hs00166163_m1 |
| PAX5 |  | Hs00277134_m1 | FYN |  | Hs00941600_m1 |
| CD28 |  | Hs00174796_m1 | MTOR |  | Hs00234508_m1 |
| CD69 |  | Hs00934033_m1 | MX1 |  | Hs00895608_m1 |
| DOCK8 |  | Hs00298892_m1 | PKC A | PRKCA | Hs00925195_m1 |
| HAVCR2 | TIM3 | Hs00958623_m1 | TNFRSF4 | OX40 | Hs00533968_m1 |
| KLRG1 |  | Rh00929962_m1 | MAPK3 | ERK1 | Hs00533968_m1 |
| STAT1 |  | Hs01013996_m1 | PDCD1 | PD1 | Hs00169472_m1 |
| STAT4 |  | Rh02896026_m1 | PIK3C2B | PI3K | Hs00898518_m1 |
| BCMA | TNFRSF17 | Hs00171292_m1 | PLCG |  | Hs01008225_m1 |
| GAPDH |  | Hs99999905_m1 | RUNX3 |  | Hs00231709_m1 |
| APOBEC3G |  | Hs00222415_m1 | SYK |  | Hs00374292_m1 |
| LAG3 |  | Hs00158563_m1 | ZAP70 |  | Hs00896345_m1 |
| LIGHT | TNFRSF14 | Hs00998604_m1 | PBX3 |  | Hs00608415_m1 |
| TACI | TNFRSF13B | Hs00963364_m1 | IL21R |  | Hs00222310_m1 |
| BAX |  | Hs00180269_m1 | RORC |  | Hs01076112_m1 |
