# Supplementary material for: Artificial Intelligence Applied to in vitro Gene Expression Testing (IVIGET) to Predict Trivalent Inactivated Influenza Vaccine Immunogenicity in HIV Infected Children
Source: Front Immunol. 2020 Oct 5;11:559590. doi: 10.3389/fimmu.2020.559590 (PMC7569088; doi:10.3389/fimmu.2020.559590)
Supplement: Supplementary Table 2 — Gene panels with probe list for T cells. [file Table_2.DOCX]

Supplementary Table 2. T cells gene panel for Fluidigm BioMark Experiment

| **Gene name** | **Gene family** | **Taqman assay ID** | **Gene name** | **Gene family** | **Taqman assay ID** |
| --- | --- | --- | --- | --- | --- |
| CXCR3 | Chemokine Receptors | Hs00171041_m1 | TGIF1 | Other | Hs00820148_g1 |
| CXCR4 | Chemokine Receptors | Hs00607978_s1 | CAMK4 | TCR-signaling | Hs00174318_m1 |
| CCR2 | Chemokines | Hs00356601_m1 | CAV1 | TCR-signaling | Hs00971716_m1 |
| CCR6 | Chemokines | Hs00171121_m1 | CD3D | TCR-signaling | Hs00174158_m1 |
| CCR7 | Chemokines | Hs00171054_m1 | CD4 | TCR-signaling | Hs00181217_m1 |
| IL21R | Cytokine Receptors | Hs00222310_m1 | DUSP4 | TCR-signaling | Hs01027785_m1 |
| IL2RA | Cytokine Receptors | Hs00166229_m1 | DUSP6 | TCR-signaling | Hs04329643_s1 |
| IL6RA | Cytokine Receptors | Hs00169842_m1 | FYN | TCR-signaling | Hs00941600_m1 |
| IL6ST | Cytokine Receptors | Hs00174360_m1 | MAPK3 | TCR-signaling | Hs00533968_m1 |
| IL7R | Cytokine Receptors | Hs00233682_m1 | NFATC1 | TCR-signaling | Hs00542678_m1 |
| IFNG | Cytokines | Hs00174143_m1 | NFKB1 | TCR-signaling | Hs00765730_m1 |
| IL10 | Cytokines | Hs00961622_m1 | PRKCA | TCR-signaling | Hs01041915_m1 |
| IL12Rb2 | Cytokines | Hs01548202_m1 | PLCG | TCR-signaling | Hs01008225_m1 |
| IL1B | Cytokines | Hs01555410_m1 | SYK | TCR-signaling | Hs00374292_m1 |
| IL2 | Cytokines | Hs00174114_m1 | ZAP70 | TCR-signaling | Rh02837378_m1 |
| IL6 | Cytokines | Hs00985639_m1 | BCL6 | TFH-associated | Hs00277037_m1 |
| FOXO3 | IFN-induced | Hs00921424_m1 | CD40L | TFH-associated | Hs00163934_m1 |
| IFIT2 | IFN-induced | Hs00533665_m1 | CXCR5 | TFH-associated | Hs00173527_m1 |
| IFNAR2 | IFN-induced | Hs01022060_m1 | ICOS | TFH-associated | Hs00359999_m1 |
| MX1 | IFN-induced | Hs00895608_m1 | ID2 | TFH-associated | Rh02796147_m1 |
| OAS1 | IFN-induced | Hs00973637_m1 | ID3 | TFH-associated | Hs00171409_m1 |
| CD38 | Immune Activation | Hs01120071_m1 | IL21 | TFH-associated | Hs00222327_m1 |
| CD69 | Immune Activation | Hs00934033_m1 | IRF4 | TFH-associated | Hs01056533_m1 |
| CD74 | Immune Activation | Hs00959498_g1 | MAF | TFH-associated | Hs00193519_m1 |
| PIK3C2B | Immune Activation | Hs00898518_m1 | PDCD1 | TFH-associated | Hs00169472_m1 |
| SOCS1 | Immune Activation | Hs00705164_s1 | PRDM1 | TFH-associated | Hs00153357_m1 |
| ABCB1 | Inflammation | Hs00184500_m1 | SELL | TFH-associated | Hs00174151_m1 |
| ADAM17 | Inflammation | Hs01041915_m1 | LIGHT | TNF Family | Hs00998604_m1 |
| CCR5 | Inflammation | Hs00152917_m1 | TNF | TNF Family | Hs00174128_m1 |
| CXCL10 | Inflammation | Hs00171042_m1 | TNFRSF4 | TNF Family | Hs00533968_m1 |
| CYBB | Inflammation | Hs00166163_m1 | TNFSF13 | TNF Family | Hs00182565_m1 |
| FAS | Inflammation | Hs00531110_m1 | BATF | Transcription Factors | Hs00232390_m1 |
| ICAM1 | Inflammation | Hs00164932_m1 | EOMES | Transcription Factors | Hs00172872_m1 |
| ITCH | Inflammation | Hs00395201_m1 | FOXP3 | Transcription Factors | Hs00203958_m1 |
| NOD2 | Inflammation | Hs01550762_g1 | GATA3 | Transcription Factors | Hs00231122_m1 |
| PTEN | Inflammation | Hs02621230_s1 | RORA | Transcription Factors | Hs00536545_m1 |
| CTLA4 | Inhibitory Receptors | Hs00175480_m1 | RORC | Transcription Factors | Hs01076112_m1 |
| HAVCR2 | Inhibitory Receptors | Hs00958623_m1 | RUNX3 | Transcription Factors | Hs00231709_m1 |
| KLRG1 | Inhibitory Receptors | Rh00929962_m1 | STAT1 | Transcription Factors | Hs01013996_m1 |
| LAG3 | Inhibitory Receptors | Hs00158563_m1 | STAT3 | Transcription Factors | Hs01047580_m1 |
| LILRB1 | Inhibitory Receptors | Hs01848117_s1 | STAT4 | Transcription Factors | Rh02896026_m1 |
| PDL1 | Inhibitory Receptors | Hs00228839_m1 | STAT5A | Transcription Factors | Rh02844611_m1 |
| TIGIT | Inhibitory Receptors | Hs00545087_m1 | TBX21 | Transcription Factors | Hs00894392_m1 |
| CD2 | Other | Hs00233515_m1 | TFAM | Transcription Factors | Hs00273372_s1 |
| DOCK8 | Other | Hs00298892_m1 | APOBEC3G | Viral restriction Factors | Hs00222415_m1 |
| MTOR | Other | Hs00234508_m1 | BST2 | Viral restriction Factors | Hs00171632_m1 |
| PTX3 | Other | Hs00173615_m1 | PRF1 | Viral restriction Factors | Hs00169473_m1 |
| TGIF1 | Other | Hs00820148_g1 | SAMHD1 | Viral restriction Factors | Hs00210019_m1 |
| CAMK4 | TCR-signaling | Hs00174318_m1 | TRIM5 | Viral restriction Factors | Hs01552559_m1 |
