# Supplementary material for: Artificial Intelligence Applied to in vitro Gene Expression Testing (IVIGET) to Predict Trivalent Inactivated Influenza Vaccine Immunogenicity in HIV Infected Children
Source: Front Immunol. 2020 Oct 5;11:559590. doi: 10.3389/fimmu.2020.559590 (PMC7569088; doi:10.3389/fimmu.2020.559590)
Supplement: Supplementary Table 3 — Single model re-analysis on top 5 ranked subsets/conditions. [file Table_3.DOCX]

**Supplementary Table 3.1 Frequency of Gene Selection for B_REM_med_stim models**

|  | | |  | |  | |  |  |
| --- | --- | --- | --- | --- | --- | --- | --- | --- |
| Variable Importance | | | Genes | | Cell Subset | | Percent | |
| 0.446971 | SELPLG_REM_stim_B | | B | | medstim | | 85.9 | |
| 0.333333 | CD69_REM_med_B | | B | | medstim | | 83.9 | |
| 0.304878 | IL2RA_REM_stim_B | | B | | medstim | | 75.8 | |
| 0.471405 | STAT4_REM_stim_B | | B | | medstim | | 74.6 | |
| 0.032506 | BCL6_REM_med_B | | B | | medstim | | 68.1 | |
| 0.396892 | IL6ST_REM_stim_B | | B | | medstim | | 65.8 | |
| 0.424114 | IL6ST_REM_med_B | | B | | medstim | | 65.2 | |
| 0.334915 | STAT4_REM_med_B | | B | | medstim | | 64.6 | |
| 0.302927 | ABCB1_REM_stim_B | | B | | medstim | | 64 | |
| 0.333333 | LIGHT_REM_med_B | | B | | medstim | | 58.3 | |
| 0.372137 | BCL2_REM_med_B | | B | | medstim | | 56.3 | |
| - | IL2RA_REM_med_B | | B | | medstim | | 54 | |
| - | BTLA_REM_med_B | | B | | medstim | | 51.9 | |
| - | IFNAR2_REM_stim_B | | B | | medstim | | 45.4 | |
| - | BCL6_REM_stim_B | | B | | medstim | | 37.3 | |
| - | IGM_REM_stim_B | | B | | medstim | | 36.6 | |

**Supplementary Table 3.2 Frequency of Gene Selection for B_DN_stim models**

|  | | |  | |  | |  |  |
| --- | --- | --- | --- | --- | --- | --- | --- | --- |
| Variable Importance | | | Genes | | Cell Subset | | Percent | |
| 0.412878 | SELPLG_DN_stim_B | | B | | stim | | 96.6 | |
| 0.299762 | HAVCR2_DN_stim_B | | B | | stim | | 93.7 | |
| 0.183829 | SAMHD1_DN_stim_B | | B | | stim | | 92.6 | |
| 0.414519 | STAT3_DN_stim_B | | B | | stim | | 91.7 | |
| 0.309909 | TLR9_DN_stim_B | | B | | stim | | 79.3 | |
| 0.180673 | PPP3CA_DN_stim_B | | B | | stim | | 66.7 | |
| 0.336767 | DUSP4_DN_stim_B | | B | | stim | | 66.6 | |
| 0.259984 | PDL1_DN_stim_B | | B | | stim | | 62.2 | |
| 0.206996 | IL2RA_DN_stim_B | | B | | stim | | 60.6 | |

**Supplementary Table 3.3 Frequency of Gene Selection for T_TFH_med_stim models**

|  | |  | |  |  | | Bootstrap Replicates | | |
| --- | --- | --- | --- | --- | --- | --- | --- | --- | --- |
| Variable Importance | | Genes | | Cell Subset | Condition | | Percent | |  |
| 0.44268 | ID2_TFH_med_T | | T | | medstim | 76.4 | |  |  |
| 0.217517 | IFNG_TFH_stim_T | | T | | medstim | 70.7 | |  |  |
| 0.410318 | IL21_TFH_stim_T | | T | | medstim | 56.3 | |  |  |
| - | CXCL10_TFH_stim_T | | T | | medstim | 45.7 | |  |  |
| - | ID2_TFH_stim_T | | T | | medstim | 45.6 | |  |  |
| - | ZAP70_TFH_stim_T | | T | | medstim | 42.5 | |  |  |
| - | TGIF1_TFH_med_T | | T | | medstim | 35.4 | |  |  |
| - | IL7R_TFH_med_T | | T | | medstim | 31.1 | |  |  |
| - | FOXP3_TFH_stim_T | | T | | medstim | 29.2 | |  |  |
| - | DUSP4_TFH_stim_T | | T | | medstim | 28.4 | |  |  |
| - | TNFRSF4_TFH_med_T | | T | | medstim | 26.5 | |  |  |
| - | PIK3C2B_TFH_stim_T | | T | | medstim | 21.9 | |  |  |
| - | CAMK4_TFH_stim_T | | T | | medstim | 20.7 | |  |  |
| - | IL2_TFH_stim_T | | T | | medstim | 17.1 | |  |  |
| - | STAT3_TFH_stim_T | | T | | medstim | 15.9 | |  |  |

**Supplementary Table 3.4 Frequency of Gene Selection for T_PBMC_med models**

|  | | |  | |  | |  | | Bootstrap Replicates | | |
| --- | --- | --- | --- | --- | --- | --- | --- | --- | --- | --- | --- |
| Variable Importance | | | Genes | | Cell Subset | | Condition | | Percent | |  |
| 0.220019 | IL6RA_PBMC_med_T | | T | | med | | 83.6 | |  |  |  |
| 0.283536 | PKC_A_PBMC_med_T | | T | | med | | 71.4 | |  |  |  |
| 0.285205 | BCL6_PBMC_med_T | | T | | med | | 71.3 | |  |  |  |
| 0.324646 | ADAM17_PBMC_med_T | | T | | med | | 66.7 | |  |  |  |
| 0.193097 | CAV1_PBMC_med_T | | T | | med | | 66.2 | |  |  |  |
| 0.219979 | GATA3_PBMC_med_T | | T | | med | | 59.4 | |  |  |  |
| 0.153223 | IL6ST_PBMC_med_T | | T | | med | | 54.1 | |  |  |  |
| 0.265554 | CCR6_PBMC_med_T | | T | | med | | 52.5 | |  |  |  |
| - | CD3D_PBMC_med_T | | T | | med | | 48.4 | |  |  |  |
| - | BST2_PBMC_med_T | | T | | med | | 47.1 | |  |  |  |
| - | CXCR4_PBMC_med_T | | T | | med | | 46.6 | |  |  |  |
| - | MAF_PBMC_med_T | | T | | med | | 45 | |  |  |  |
| - | IL21R_PBMC_med_T | | T | | med | | 32.4 | |  |  |  |
| - | IFNG_PBMC_med_T | | T | | med | | 31.6 | |  |  |  |
| - | IRF4_PBMC_med_T | | T | | med | | 28.9 | |  |  |  |
| - | ICOS_PBMC_med_T | | T | | med | | 25.8 | |  |  |  |
| - | PTX3_PBMC_med_T | | T | | med | | 14.5 | |  |  |  |
| - | ID2_PBMC_med_T | | T | | med | | 4.7 | |  |  |  |

**Supplementary Table 3.5 Frequency of Gene Selection for B_DN_med_stim models**

|  | | |  | |  | |  | | Bootstrap Replicates | | |
| --- | --- | --- | --- | --- | --- | --- | --- | --- | --- | --- | --- |
| Variable Importance | | | Genes | | Cell Subset | | Condition | | Percent | |  |
| 0.101507 | SELPLG_DN_stim_B | | B | | medstim | | 99.2 | |  |  |  |
| 0.402705 | STAT3_DN_stim_B | | B | | medstim | | 96.5 | |  |  |  |
| 0.315574 | HAVCR2_DN_stim_B | | B | | medstim | | 80 | |  |  |  |
| 0.371186 | TNFSF13_DN_med_B | | B | | medstim | | 79.1 | |  |  |  |
| 0.372813 | DUSP4_DN_stim_B | | B | | medstim | | 76.5 | |  |  |  |
| 0.043288 | SAMHD1_DN_stim_B | | B | | medstim | | 76.1 | |  |  |  |
| 0.223812 | TLR9_DN_stim_B | | B | | medstim | | 75.9 | |  |  |  |
| 0.024006 | APOBEC3G_DN_med_B | | B | | medstim | | 72.3 | |  |  |  |
| 0.224184 | GATA3_DN_med_B | | B | | medstim | | 64.6 | |  |  |  |
| 0.315955 | PPP3CA_DN_stim_B | | B | | medstim | | 58.2 | |  |  |  |
| 0.292493 | IFIT2_DN_med_B | | B | | medstim | | 55.2 | |  |  |  |
| 0 | PDL1_DN_stim_B | | B | | medstim | | 50.7 | |  |  |  |
| 0.228588 | IL2RA_DN_stim_B | | B | | medstim | | 50.4 | |  |  |  |
| - | MX1_DN_med_B | | B | | medstim | | 43 | |  |  |  |
